# Conditional Survival Analysis Demonstrates that Recurrence Risk of Surgically Treated Hepatocellular Carcinoma Evolves with Time

**DOI:** 10.1007/s11605-017-3437-7

**Published:** 2017-05-23

**Authors:** Yong Keun Park, Sung Kyu Song, Bong-Wan Kim, Seung-Keun Park, Jong In Lee, Seung Su Lim, Hee-Jung Wang

**Affiliations:** 1 Department of Surgery, Ajou University School of Medicine, 164 World cup-ro, Yeongtong-gu, Suwon, 16499 South Korea; 2 Department of Surgery, Catholic Kwandong University International St. Mary’s Hospital, Incheon, South Korea; 3 Division of SMEs Innovation, Supercomputing Modeling and Simulation Center, Department of Supercomputing M&S Technology Development, Korea Institute of Science and Technology Information, Daejeon, South Korea; 4 Graduate School of Public Health, Ajou University, Suwon, South Korea

**Keywords:** Conditional survival, Hepatectomy, Survival analysis, Tumor recurrence

## Abstract

**Objective:**

The study aim was to investigate long-term change in tumor recurrence risk in patients with hepatocellular carcinoma (HCC) after hepatic resection. Recurrence probability over time was estimated by conditional survival (CS) analysis.

**Patients and Methods:**

Early-stage HCC patients with hepatic resection were selected for inclusion from our surgery database. Variables predictive of tumor recurrence were identified by univariate and multivariate analyses. Five-year recurrence-free CS probability was calculated for all patients and for risk groups stratified by independent predictors.

**Results:**

In this series of 436 patients, tumor size >5 cm, microvascular invasion, positive resection margin, liver cirrhosis, and a indocyanine green retention ratio at 15 min (ICG-R15) >20% were independently predictive of tumor recurrence. The estimated 5-year recurrence-free CS probability improved with each additional year of recurrence-free survival, and the improvement was significantly greater in the high-risk than in the low- or intermediate-risk groups.

**Conclusion:**

CS provides added value during follow-up of early-stage HCC patients treated by surgical resection.

## Introduction

Hepatocellular carcinoma (HCC) is a frequently diagnosed cancer and a leading cause of cancer-related deaths worldwide.^1^ Epidemiological evidence confirms that both HCC incidence and mortality increased between 2000 and 2010.^2^ Surveillance of high-risk groups helps in the early detection of HCC, thus making curative treatment more likely. Hepatic resection is considered a curative treatment for early-stage HCC, especially for cases of solitary tumors and compensated liver function,^3^ but the high rate of tumor recurrence is a major shortcoming and the main cause of poor outcomes.^4^^,^ 5

The accurate prediction of oncologic prognosis and the probability of tumor recurrence are important for planning follow-up surveillance and additional treatment of *HCC* patients. Studies evaluating patients stratified by various predictors of recurrence risk^6^^,^ 7 have identified microvascular invasion (McVI) as a factor that can affect the prognosis of postoperative recurrence. Subsequent studies have focused on preoperative prediction of McVI.^8^^,^ 9 Cirrhosis has also been associated with increased risk of tumor recurrence after curative treatment.^10^

Although McVI and cirrhosis have prognostic value for clinicians and patients, in the immediate postoperative period, there is little evidence that they are useful to forecast the probability of recurrence after a long tumor-free period. Patients at high risk of tumor recurrence who remain recurrence-free for long periods may not have the same risk as that at the time of surgery. Different prediction tools or methods may be needed to provide an insight into change in prognosis over time. This need has been addressed recently using the concept of conditional survival (CS) analysis.^11^^,^ 12

CS analysis estimates the probability of surviving a chronic disease for an additional *t* years, given a history of survival for *s* years.^13^^,^ 14 Adjusting for the time (*s* years), the patient has already survived serves to increase accuracy of the estimated prognosis for the next *t* years. Applying this concept to recurrence-free survival, we can also get the information of recurrence-free CS. Applying CS analysis to recurrence-free survival, it can be estimated how much the risk of tumor recurrence evolves over time and whether the predictive power of well-known risk factors changes. Despite the potential benefits, no previous studies have used CS analysis to evaluate long-term HCC survival. This retrospective study analyzed recurrence-free CS in patients with hepatic resection for early-stage HCC and assessed independent risk factors of tumor recurrence at initial presentation and changes in their predictive powers over a long recurrence-free period.

## Patients and Methods

This retrospective analysis was conducted in a series of patients with hepatic resection for HCC at the department of surgery in our institute between 1994 and 2011. Patients with radiologically confirmed solitary HCC without macrovascular invasion were defined as “early stage and curatively resectable,”^15^ and were eligible for inclusion. Patients with multiple tumors, ruptured tumors, tumors with preoperatively identified macrovascular invasion, lymph node metastasis, and/or distant, including adrenal, metastasis were excluded. In all patients, the diagnosis of HCC had been pathologically confirmed. Cirrhosis was histopathologically confirmed as METAVIR stage 4. Patient data were extracted from prospectively collected medical records, which included demographics, the etiology of underlying liver disease, pathologic findings of the specimen, surgical results, and oncologic outcomes. This study was approved by the Medical Center Institutional Review Board, along with a waiver of informed consent.

Recurrence-free CS probability was estimated from Kaplan–Meier cumulative survival data. *S*(*t*) indicates the actuarial life table survival at time *t*, and CS indicates the probability that a patient will survive an additional y years given a survival history of *x* years,^11^ and is calculated as CS (*y*|*x*) = *S*(*x* + *y*)/*S*(*x*). For example, the conditional probability of a patient who has already survived 4 years of surviving an additional year, *S*(1/4), is calculated by dividing the 5-year actuarial life table survival estimate, *S*(5), by the 4-year survival estimate, *S*(4); thus, *S*(1/4) = *S*(1 + 4)/*S*(4) = *S*(5)/*S*(4).

The decision to perform hepatic resection and the extent of the resection were based on assessment of the patient’s general condition, Child-Pugh liver function score, and indocyanine green retention rate at 15 min (ICG-R15). If the ICG-R15 value was favorable, hepatic resection was considered even when the Child-Pugh class was B. An additional prediction scoring system was used to select patients for major resection.^16^ The majority of hepatic resections were intended to completely remove the portal territory of the tumor-containing region to be anatomical resection. Major resection was defined as the resection of three or more segments following the Brisbane classification^17^; minor resection involved two segments or less. If anatomical resection was not possible, we tried to obtain an appropriate margin length. Intraoperative ultrasonography was used to identify any additional lesions and to determine the optimal resection plane. Hospital mortality included inpatient deaths that were associated with hepatic resection.

After discharge, all patients were followed every 1 to 3 months during the first 2 years and every 3 months for the following 3 years. Thereafter, patients were seen twice a year. Liver function tests and serum α-fetoprotein (AFP) levels were obtained at every visit, and routine surveillance included abdominal ultrasonography or computerized tomography (CT) scans and chest radiographs every 3 months during the first 2 years and every 6 months thereafter.

Recurrence was defined as the presence of a radiologically confirmed tumor with an increase in serum AFP. If a recurrence was highly suspected without any clear imaging evidence, hepatic arteriography and lipiodol CT scans were performed. Depending on the tumor status and underlying liver function, patients with intrahepatic recurrence were treated by re-resection, local ablation, transcatheter arterial chemoembolization (TACE), or salvage liver transplantation (SLT). Treatment options for patients with extrahepatic metastatic disease included metastasectomy, TACE, radiation therapy, and targeted therapy with sorafenib.

### Statistical Analysis

All continuous variables were expressed as mean ± standard deviation or median (minimum-maximum range). Data were analyzed using SPSS 18.0 for Windows (SPSS, Chicago, IL, USA). Cumulative survival was estimated by the Kaplan–Meier method, and the rates were compared using the log rank test. Multivariate analysis and the Cox regression proportional hazards model were used to identify factors that independently predicted recurrence-free survival. Variables significantly related to survival in the Kaplan–Meier analysis were used to stratify the study population by risk of recurrence after hepatic resection, and the Kaplan–Meier survival data were used to estimate 5-year CS. *P* values <0.05 were considered statistically significant.

## Results

**Fig. 1:**
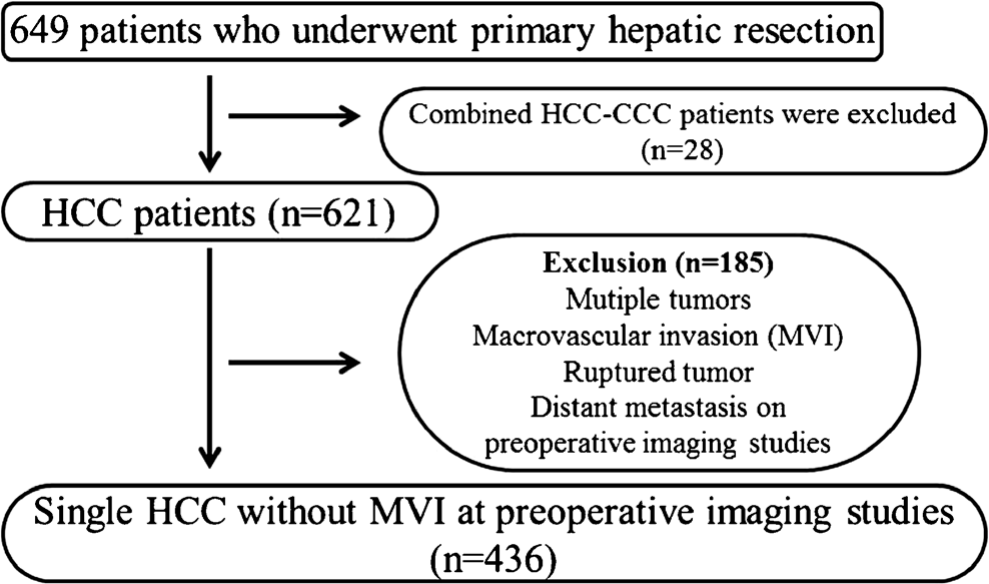
Flow diagram showing the selection of patients who were eligible for this study. *HCC* hepatocellular carcinoma, *CCC* cholangiocarcionoma

A total of 649 patients experienced primary hepatic resection for HCC during the study period. Twenty-eight were diagnosed with combined HCC and intrahepatic-cholangiocarcinoma and 185 with multiple tumors, ruptured tumors, macrovascular invasion, and/or distant metastasis including adrenal metastasis discovered during preoperative evaluation were excluded (Fig. 1). The characteristics of the remaining 436 patients with solitary HCC and no macrovascular invasion on preoperative imaging are summarized in Table 1. There was a preponderance of male patients (340, 78%); the median age was 52 years (range 20–76 years), 324 patients (74.3%) tested positive for hepatitis B surface antigen, and 26 (6.0%) tested positive for hepatitis C virus. Four hundred and fourteen patients (95.2%) were Child-Pugh class A and 19 (4.4%) were class *B. Major* hepatic resection of three or more Couinaud’s segments was performed in 129 patients (29.6%).

**Table 1:** Univariate and multivariate analysis to identify prognostic factors associated with tumor recurrence

Factors	Number of patients	MDFST^a^ (95% CI)	*P* value	Odds ratio (95% CI)	*P* value
*Clinical factors*					
Gender			*0.694*		
Male	340	52.5 ± 5.82 (41.09–63.91)			
Female	96	37.57 ± 7.56 (22.75–52.40)			
Age			*0.915*		
≤65	387	51.0 ± 5.81 (39.60–62.40)			
>65	49	37.5 ± 3.00 (31.62–43.38)			
Hepatitis B or C infection status			*0.08*		
No	89	72.87 ± 22.26 (29.25–116.49)			
Yes	347	43.93 ± 6.20 (31.76–56.10)			
Child-Pugh classification			*0.268*		
A	415	52.0 ± 6.21 (39.82–64.18)			
B or C	19	37.37 ± 15.08 (7.81–66.93)			
Alpha-fetoprotein (ng/L)			*0.022*		
≤9	139	56.53 ± 21.55 (14.29–98.77)			
>9	291	43.93 ± 7.72 (28.80–59.06)			
ALT (IU/dL)			*0.637*		
≤40	225	63.0 ± 11.70 (40.07–85.94)			
>40	210	43.30 ± 5.44 (32.64–53.96)			
ICGR15 (%)			*0.001*	1.829 (1.292–2.588)	*0.001*
≤20	350	58.13 ± 8.23 (41.99–74.27)			
>20	72	29.0 ± 5.45 (18.31–39.69)			
Preoperative TACE			*0.857*		
No	279	52.5 ± 8.13 (36.57–68.44)			
Yes	137	45.5 ± 7.82 (30.18–60.83)			
*Pathologic factors*					
Size of tumor (cm)			*0.002*	1.867 (1.334–2.613)	*<0.001*
≤5	316	58.0 ± 6.80 (44.68–71.32)			
>5	120	22.2 ± 3.46 (15.43–28.97)			
Microvascular invasion			*<0.001*	1.891 (1.397–2.560)	*<0.001*
Absent	262	72.87 ± 14.56 (44.34–101.40)			
Present	174	24.10 ± 3.20 (17.83–30.37)			
Macrovascular invasion			*<0.001*		
No	388	53.0 ± 6.39 (40.47–65.53)			
Yes	48	16.0 ± 5.02 (6.17–25.83)			
Intrahepatic metastasis			*0.005*		
No	375	53.0 ± 6.42 (40.41–65.59)			
Yes	61	20.50 ± 7.42 (5.99–35.00)			
Histologic grading by Edmondson and Steiner’s classification			*0.016*		
I–II	174	66.30 ± 12.22 (42.37–90.29)			
III or IV	223	37.57 ± 5.88 (26.05–49.09)			
Extent of resection			*0.639*		
Major	129	52.00 ± 16.50 (19.67–84.33)			
Minor	307	49.67 ± 6.47 (36.99–62.34)			
Microscopic resection margin			*<0.001*	1.915 (1.178–3.113)	*0.009*
Negative	397	53.00 ± 6.30 (40.65–65.36)			
Positive	34	14.00 ± 2.94 (8.25–19.75)			
Cirrhosis^b^			*<0.001*	1.74 (1.285–2.356)	*<0.001*
No	199	86.00 ± 19.06 (48.64–123.36)			
Yes	212	37.37 ± 5.30 (26.98–47.76)			
Japanese TNM stage			*<0.001*		
I	68	60.40 ± 10.19 (40.42–80.38)			
II	285	56.53 ± 9.26 (38.37–74.69)			
III	67	27.73 ± 10.38 (7.39–48.07)			
IVA	16	6.00 ± 1.26 (3.54–8.46)			

*CI* confidence interval

^a^Median disease-free survival time (month)

^b^Cirrhosis was defined as METAVIR fibrosis stage 4

**Fig. 2:**
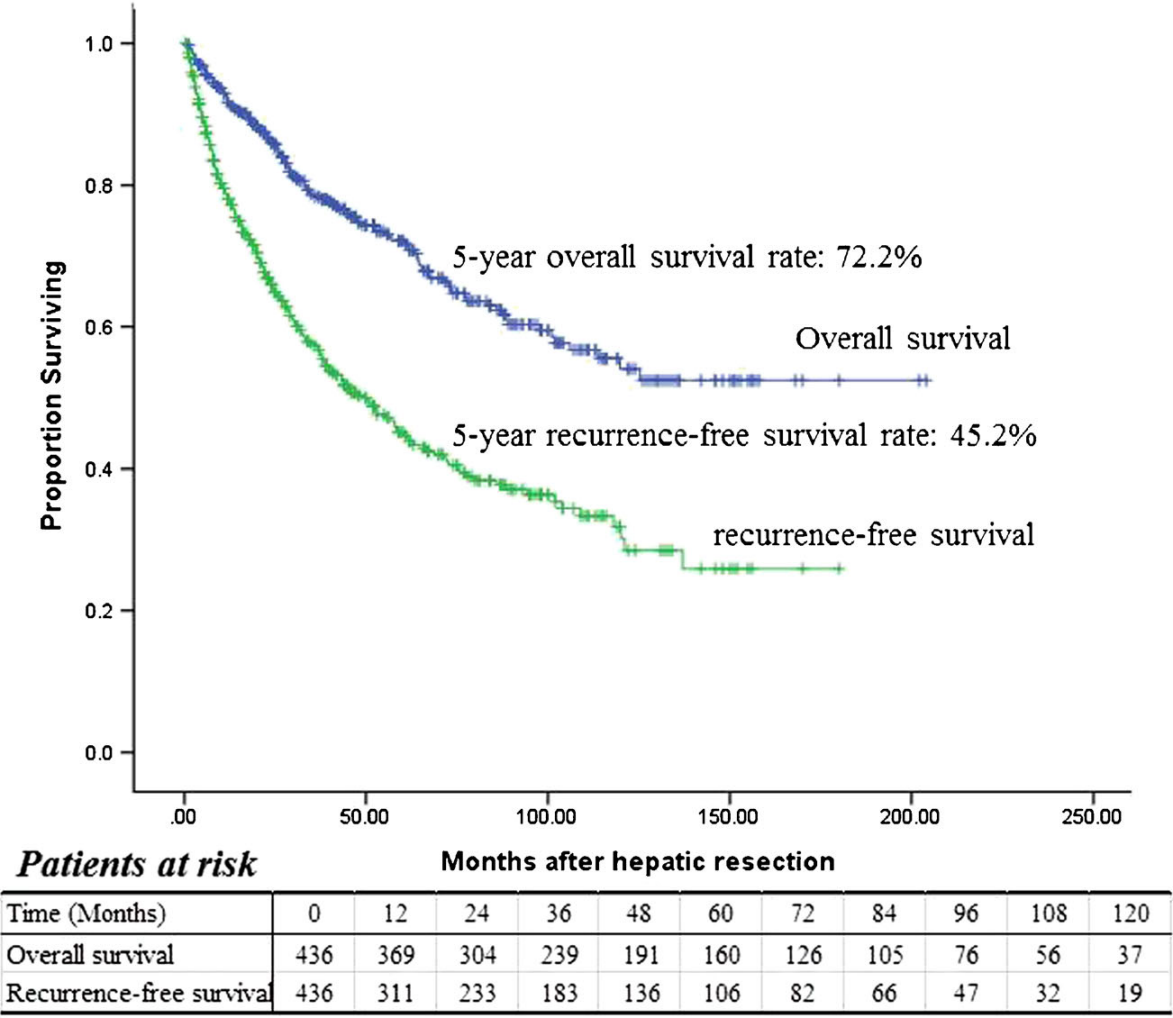
Cumulative recurrence-free and overall survival of the 436 patients with resected early-stage HCC estimated by the Kaplan–Meier method

The median follow-up was 41.3 months (range 1–186). At the time of data collection, 127 patients (19.6%) had died. Four (0.92%) patients died of preoperative complications within the same hospitalization period; one of the four cases was given an SLT but did not recover. Eight died of non-liver-related causes, including another type of cancer, pneumonia, or heart attack. Twelve died of advanced liver cirrhosis, four of whom had tumor recurrence. The remaining 103 patients (83.7%) died following tumor recurrence. At the time of analysis, 342 patients were alive, and of those, 192 had not experienced a recurrence; 117 had recurrences. Median overall survival was 41 months (range 1–204); the 5-year survival rate was 72.2% (Fig. 2).

At the time of data collection, a total of 224 patients, including both those who were alive and those who had died, had experienced tumor recurrences. The median time to recurrence was 26 months (range 1–180). The rate of recurrence-free survival was 66.0% at 2 years and 45.2% at 5 years (Fig. 2). Of the 224 tumor recurrences, 92 (41.1%) occurred within the first year and 137 (61.2%) occurred within 24 months of the initial hepatic resection. Fifty (22.3%) tumor recurrences occurred between the second and fifth postoperative years; the remaining 37 (16.5%) occurred more than 5 years after hepatic resection. The patterns of tumor recurrence differed depending on when they occurred. Of the recurrences that occurred within 24 months after the initial hepatic resection, 55% were solitary or oligonodular intrahepatic lesions and 45% were diffuse intrahepatic or distant metastatic lesions. The types of tumor recurrence that occurred after 24 months were either solitary or oligonodular intrahepatic lesions (80.6%) or diffuse intrahepatic or distant metastatic lesions (19.4%, *P* = 0.002).

**Fig. 3:**
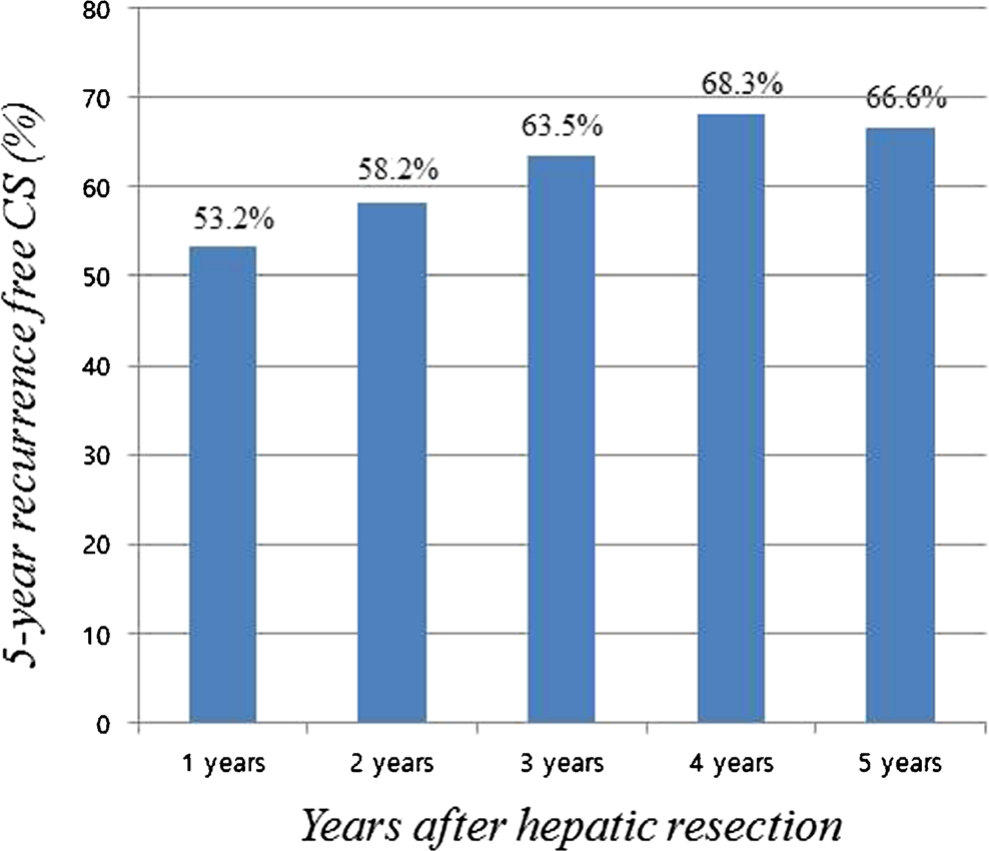
Five-year recurrence-free conditional survival (CS) at each additional year after hepatic resection

**Fig. 4:**
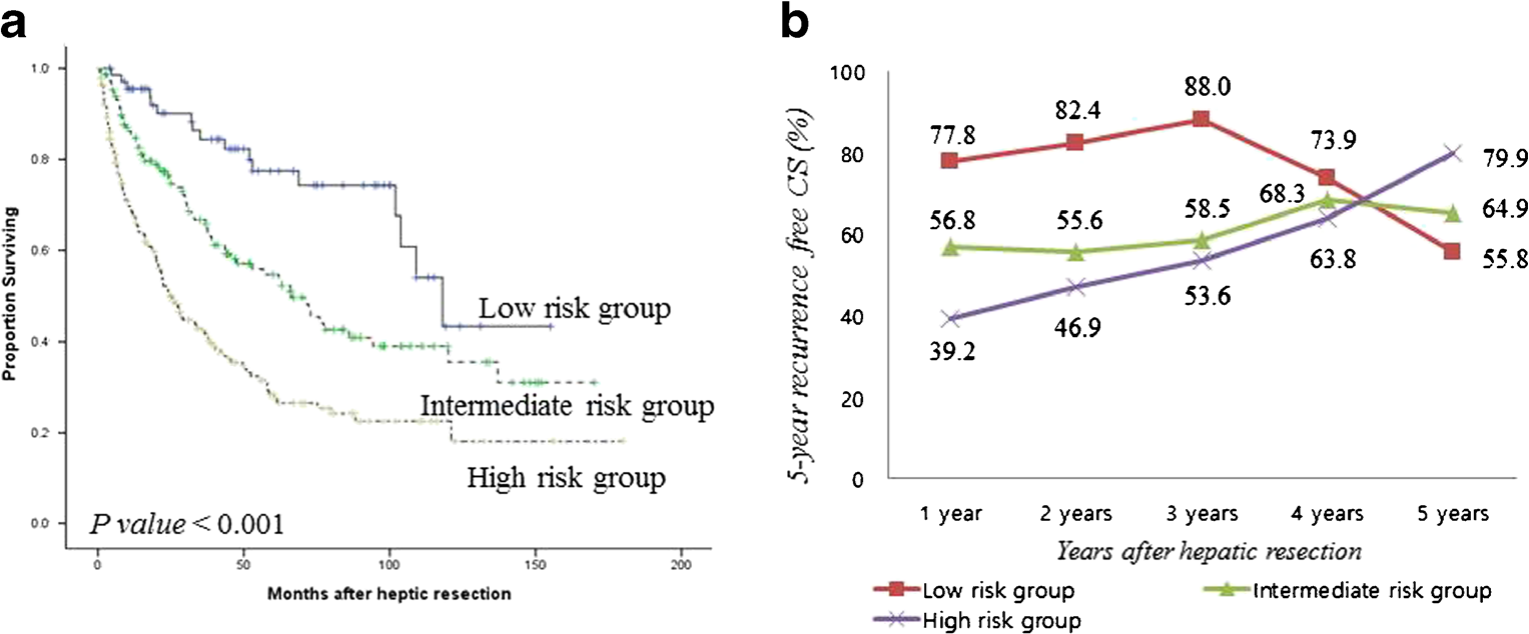
Recurrence-free survival curves (**a**) and five-year recurrence-free CS probability (**b**) in three risk groups. The low-risk group had no independent predictors, the intermediate-risk group had one predictor, and the high-risk group had two or more predictors

Ten-year recurrence-free survival data were used to calculate CS probability. Figure 3 shows the 5-year recurrence-free CS probability and the changes in CS with each additional year without recurrence. The 5-year recurrence-free CS probability increased by 14.5%, from 58.2 to 66.6% between 2 and 5 years after hepatic resection. The univariate predictors of tumor recurrence are listed in Table 1. Multivariate analysis revealed five predictors that were independently associated with tumor recurrence. These were ICG-R15 >20%, tumor size >5 cm, presence of microvascular invasion, positive microscopic resection margin, and background liver cirrhosis (Table 1). Based on the multivariate analysis results, patients were stratified by low (having no predictor), intermediate (having one predictor), and high (having two or more predictors) recurrence risk. Figure 4a shows the recurrence-free survival in the three risk groups, which had significantly different prognoses (*P* < 0.001). Figure 4b shows the estimated 5-year recurrence-free CS probability and the changes in CS in each risk group with each additional year of recurrence-free survival. Interestingly, a favorable 70.5% increase in 5-year recurrence-free probability occurred in the high-risk group between the second (46.8%) and fifth (79.8%) years after hepatic resection. In the low or intermediate risk groups, there were no significant changes in CS probability over time, but the 5-year recurrence-free CS probability in the low risk group decreased from 82.3% in year 2 to 55.8% in year 5. Given the recurrence-free survival in the 4 years after hepatic resection, the resulting 5-year CS recurrence-free probability was nearly identical or even reversed between the risk groups.

**Fig. 5:**
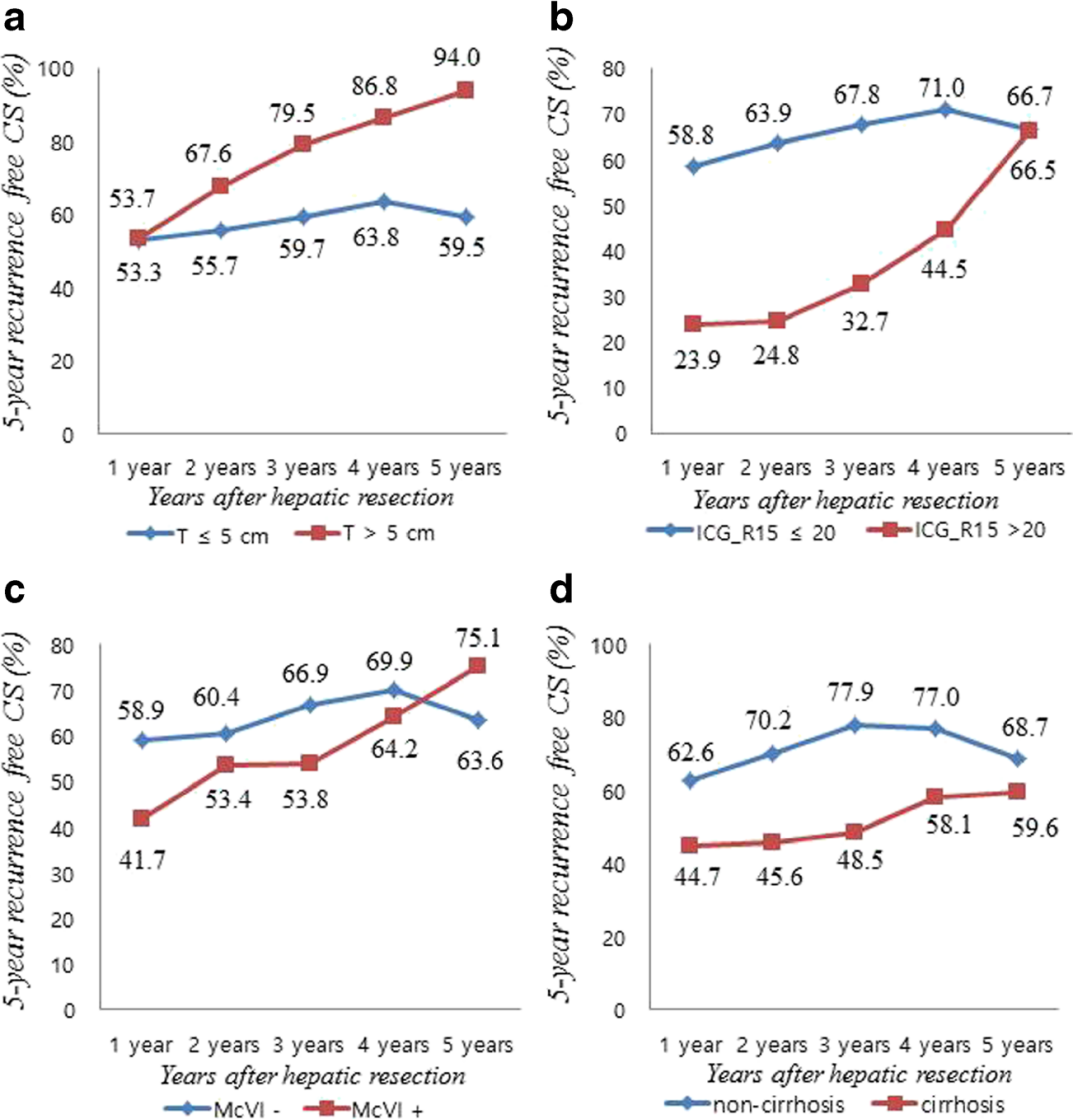
Five-year recurrence-free CS probability with **a** tumor size less than or greater than 5 cm, **b** ICG-R15 values less than or more than 20%, **c** presence or absence of microvascular invasion, and **d** presence or absence of background liver cirrhosis

The adjusted 5-year recurrence-free CS probability was calculated for patients with or without an independent prognostic factor at hepatic resection. For patients with tumors <5 cm, the 5-year recurrence-free CS probabilities were 53.3% at year 1 and 59.5% at year 5. For patients with tumors >5 cm, the corresponding CS probabilities were 53.7 and 94.0%. The increase in 5-year recurrence-free CS probability was thus much greater in patients with large tumors (a 75% increase from 53.7 to 94.0%) than in those with small tumors (an 11.6% increase from 53.3 to 59.5%, Fig. 5a). The differences in recurrence probability associated with preoperative ICG-R15 above or below 20% and by the absence or presence of microvascular invasion were similar to those seen for tumor size. In patients stratified by ICG-R15, the 1-year probabilities of recurrence were 58.8 and 23.9%, but by 5 years, the difference (66.7 vs. 66.5%) had nearly disappeared. The increase in 5-year recurrence-free CS probability was thus much larger in patients with preoperative ICG-R15 values >20% (a 178% increase from 23.9 to 66.5%) than in those with values <20% (a 13% increase from 58.8 to 66.7%, Fig. 5b) The increase in 5-year recurrence-free CS probability in patients with microvascular invasion was 82.2% (from 41.7 to 75.1%) and 16.5% (from 58.9 to 63.6%) in those without microvascular invasion (Fig. 5c). However, the trend in change in 5-year recurrence-free CS probability was different in patients with or without background liver cirrhosis. The 5-year recurrence-free CS probability was higher in patients without baseline cirrhosis than in those with it. The increases in 5-year recurrence-free CS probability from the first to fifth years in the two groups were not significantly different. The increase in the non-cirrhosis group was 9.7% (62.6 to 68.7%) and 33% in the cirrhosis group (44.7 to 59.6%, Fig. 5d).

## Discussion

*Hepatic resection is a potentially curative treatment of early-stage HCC in patients with preserved liver function*. With considerable improvements in the selection process and intra- and preoperative care, mortality rates have significantly decreased.^18^ The high rate of postoperative tumor recurrence remains a major problem, and many studies have focused on predicting the risk of tumor recurrence.^6^^,^ 7 ^,^ 19 ^,^ 20 The values of parameters evaluated in previous studies for prediction of recurrence were obtained around the time of surgery, and thus, could not provide information about changes in postoperative oncologic risks. We generally assume that risk of recurrence would decrease, but the supporting evidence is weak. None of the previous studies have accounted for the effect of recurrence-free survivorship on the evolving risk of tumor recurrence in HCC.

In this study, we evaluated changes in risk over time in early-stage patients with good liver function reserve and who were recommended for hepatic resection according to the Barcelona Clinic Liver Cancer (BCLC) group staging system.^15^ We classified the patients into three groups based on independent risk factors that were identified by multivariate analysis. As expected, each group had significantly different prognoses during the immediate postoperative period. However, the differences among survivors become smaller over time. Patients with unfavorable risk factors (high-risk group) had greater improvements in recurrence-free CS over time. If these patients were recurrence-free in the first 3 years after hepatic resection, then their chance of remaining recurrence-free in the next 5 years increased dramatically to more than 50%. The calculated CS change in recurrence probability between 2 and 5 years after hepatic resection increased by 70.5% from 46.8 to 79.8% (Fig. 4b).

Estimates of oncologic outcomes are generally presented as 5-year recurrence-free or overall survival at the time of initial curative treatment. These survival data are useful for counseling patients at their initial visit or for comparing outcomes of various therapeutic modalities. However, they provide little information about changes in risk over time. For cancers with clinically significant high rates of early recurrence, conventional recurrence-free survival estimates are only applicable at initial presentation and become inaccurate and meaningless over time. Previously, even after remaining recurrence-free for a specific time, patients at high risk of recurrence at initial presentation were still considered to have a high probability of tumor recurrence or mortality. Knowledge of how risk profiles change over time would provide more precise information on current disease status and prognosis. CS estimates provide dynamic prognostic information through data analysis considering how long a patient has survived after the initial diagnosis. CS analysis uses Kaplan–Meier analysis of baseline data or actuarial life table survival estimates and does not require any additional data, unjustified assumptions, or specialized methods.^14^ Additionally, CS accounts for changes in hazard rates over time as well as dynamic changes in prognosis. If CS probability in high-risk groups stratified by a given prognostic factor improves or becomes similar to that of a low-risk group, it can be cautiously assumed that the negative prognostic effect of the factor has disappeared. In this series, that was observed in patients stratified by tumor size >5 cm at 1 year, McVI at 4 years, and ICG-R15 >20 at 5 years (Fig. 5). Changes in CS estimates might be used to guide clinical decision-making with respect to the recurrence surveillance schedule and the need for long-term follow-up.

Previous studies have investigated changes in the risk of cancer-related deaths over a given survival period in various cancers. High-risk melanoma patients were found to have the same likelihood of cancer-related deaths as low-risk patients after 8 years of survival, even though the predicted 10-year survival rates were substantially worse in high- than in low-risk patients at initial presentation.^21^ In pancreatic cancer patients, 3-year cancer-specific CS increased dramatically in the 3 years after diagnosis, with a larger relative improvement in CS over time in patients with advanced-stage cancer.^22^ CS in patients with head and neck cancer found that the difference in prognoses between early- and advanced-stage patients narrowed as time elapsed after diagnosis.^23^ We did not evaluate overall CS, but rather recurrence-free CS, to estimate the change in oncologic risk in HCC patients because underlying liver disease can cause death, leading to bias in the assessment of tumor-specific characteristics. In fact, some HCC patients in this series died of advanced liver disease, not only from tumor progression. Consequently, using recurrence-free CS seemed to be a more accurate method of estimating changes in oncologic risks in early HCC.

Recurrence patterns differed depending on the time of recurrence. When tumor recurrence occurred 2 years postoperatively, over 80% were solitary or oligonodular intrahepatic lesions that could be treated using various therapies including liver transplantation, re-resection, or local ablation. A possible explanation is that late recurrences may not be true recurrences of the initial mass, but rather a newly occurring de novo malignancy within the etiologic context of liver disease. In this study, we found that background liver cirrhosis was an independent predictor of tumor recurrence. Throughout the study period, the 5-year recurrence-free CS probability was higher in patients without than with cirrhosis. The increases of 5-year recurrence-free CS probability in the two groups were not significantly different. These results indicate a different trend for cirrhosis compared to the effects of other independent risk factors and lead to the reasonable hypothesis that underlying liver disease has a constant effect on tumor recurrence beginning in the early period and continuing after hepatic resection for HCC. Other parameters may have a strong effect on recurrence risk only in the early period. The results of this study are consistent with those of previous studies that found cirrhosis to be a unique risk factor for late tumor recurrence after hepatic resection.^24^ More detailed study is needed to clarify the influence of cirrhosis in HCC prognosis.

Recent advances in cancer biology, including genomics, have allowed for an increasingly molecular approach to HCC diagnosis, evaluation of prognosis, and treatment choices.^20^^,^ 25 Estimation of the prognosis of individual patients has been applied in various cancers and has been highly predictive. However, the prognostic value of estimates of individual risk of tumor recurrence based on molecular markers at initial presentation may not be valid after the passage of time. Consequently, it is necessary to assess how each molecular predictor contributes to estimates by CS analysis, which may have more predictive power.

The study limitations include its retrospective design, even though the data were collected prospectively. Furthermore, we did not include information on postoperative patient status, such as the use of antiviral agents, progression of underlying liver disease, or exposure to other oncologic risks in the analysis. Such events can influence tumor recurrence or de novo malignancy. External validation of these findings using a multicenter database is required.

## Conclusions

This study showed that CS probability can be more predictive of continuing freedom from recurrence in long-term early-stage HCC survivors than conventional methods of survival prediction applied in the immediate postoperative period. The initial recurrence-free survival expectancy improved more substantially in patients at high risk of tumor recurrence than in those at low or intermediate risk. Such results allow provision of important baseline information for counseling and scheduling of recurrence surveillance after hepatic resection in patients with early-stage HCC.
